# The versatile X-ray beamline of the Munich Compact Light Source: design, instrumentation and applications

**DOI:** 10.1107/S1600577520008309

**Published:** 2020-07-31

**Authors:** Benedikt Günther, Regine Gradl, Christoph Jud, Elena Eggl, Juanjuan Huang, Stephanie Kulpe, Klaus Achterhold, Bernhard Gleich, Martin Dierolf, Franz Pfeiffer

**Affiliations:** a Department of Physics, Technical University of Munich, James-Franck-Straße 1, 85748 Garching, Germany; b Munich School of BioEngineering, Technical University of Munich, Boltzmannstraße 11, 85748 Garching, Germany; c Department of Diagnostic and Interventional Radiology, Klinikum rechts der Isar, Technical University of Munich, Ismaninger Straße 22, 81675 Munich, Germany

**Keywords:** Munich Compact Light Source, inverse Compton X-ray sources, X-ray phase-contrast imaging and tomography, X-ray absorption spectroscopy, micro-beam radiation therapy

## Abstract

Inverse Compton scattering provides means to generate low-divergence partially coherent quasi-monochromatic, *i.e.* synchrotron-like, X-ray radiation on a laboratory scale. This enables the transfer of synchrotron techniques into university or industrial environments. Here, the Munich Compact Light Source is presented, which is such a compact synchrotron radiation facility based on an inverse Compton X-ray source (ICS). The recent improvements of the ICS are reported first and then the various experimental techniques which are most suited to the ICS installed at the Technical University of Munich are reviewed. For the latter, a multipurpose X-ray application beamline with two end-stations was designed. The beamline’s design and geometry are presented in detail including the different set-ups as well as the available detector options. Application examples of the classes of experiments that can be performed are summarized afterwards. Among them are dynamic *in vivo* respiratory imaging, propagation-based phase-contrast imaging, grating-based phase-contrast imaging, X-ray microtomography, *K*-edge subtraction imaging and X-ray spectroscopy. Finally, plans to upgrade the beamline in order to enhance its capabilities are discussed.

## Abstract 2

The multipurpose X-ray beamline of the Munich Compact Light Source, a compact synchrotron radiation facility based on an inverse Compton X-ray source, is presented including its design and instrumentation. Its wide range of capabilities is discussed based on application examples ranging from spectroscopic as well as phase-contrast X-ray imaging and tomography to X-ray absorption spectroscopy or micro-beam radiation therapy research.

## 1. Introduction   

During the last decades, large-scale synchrotron facilities have been the driving force in the development of new X-ray imaging techniques that overcome the limits of conventional absorption imaging. Although many of these techniques, *e.g.* *K*-edge subtraction imaging (Thomlinson *et al.*, 2018 ▸) or phase-contrast imaging (Endrizzi, 2018 ▸), provide benefits for medical X-ray imaging, most of them have not been implemented in clinical routines yet. This is mostly because they rely on X-ray properties which are – in contrast to conventional X-ray tubes – only provided by synchrotron radiation like (partial) coherence and/or high brilliance. Accordingly, there is substantial interest in X-ray sources that are capable of producing such radiation, but only require a small footprint and low expenses for operation as well as maintenance. Liquid-metal jet X-ray sources (Hemberg *et al.*, 2003 ▸; Otendal *et al.*, 2008 ▸; Larsson *et al.*, 2011 ▸) fulfil these requirements, but only at the *K*-lines of the material employed in the alloys (mainly indium, gallium and tin). Therefore, high brilliance at these sources is limited to a comparably low X-ray energy of 9.2 keV (gallium) and with restrictions at a higher energy of 24.2 keV (indium). This issue of just fixed, relatively low X-ray energies (lines) with high brilliance can be overcome by inverse Compton scattering of a low-energy photon (typically visible light or infrared light) at a relativistic electron. The energy of the X-rays created in this process scales quadratically with electron energy and linearly with photon energy (Milburn, 1963 ▸; Arutyunian & Tumanian, 1963 ▸), and can thus usually be changed freely by adjusting the electron energy. Laser-electron scattering was experimentally demonstrated in the 1960s in proof-of-principle experiments (*e.g.* Fiocco & Thompson, 1963 ▸; Kulikov *et al.*, 1964 ▸; Bemporad *et al.*, 1965 ▸; Ballam *et al.*, 1969 ▸; Kulikov *et al.*, 1969 ▸). Nevertheless, it took a long time until the first γ-ray sources emerged in the late 1970s and 1980s (Federici *et al.*, 1980 ▸; Sandorfi *et al.*, 1983 ▸; Yamazaki *et al.*, 1985 ▸; Kezerashvili *et al.*, 1993 ▸; D’Angelo *et al.*, 2000 ▸). Even later, compact inverse Compton (X-ray) sources (ICSs) were (re-)considered at the end of the 1980s to early 1990s (Sprangle *et al.*, 1989 ▸, 1992 ▸; Carroll *et al.*, 1990 ▸; Tompkins *et al.*, 1993 ▸; Blum, 1993 ▸; Chen *et al.*, 1994 ▸) after both laser- and electron accelerator technology had matured. Nevertheless, their flux has only recently reached levels suitable for applications. This further stimulated the development of ICSs resulting in a steadily increasing number of ICSs operating, under construction or being designed.

Two different concepts exist: either the ICSs are based on an electron storage ring coupled to a laser (ring) resonator or they are based on a linear accelerator coupled to a low-repetition-rate high-peak-power laser system. Optionally, the latter can be equipped with a laser recirculator or ring-down cavity in order to artificially match the laser repetition rate at the interaction point to the one of the linear accelerator which can be either a super-conducting one or a conventional one. On the one hand, super-conducting linear accelerators support much higher electron pulse rates enabling higher interaction frequencies and thus X-ray flux. But on the other hand the system costs and complexity increase significantly. Therefore, many of the linear-accelerator-based ICSs operating or under construction as an X-ray (user) facility rely on a conventional accelerator, *e.g.* Tsinghua Thomson X-ray source (TTX) (Du *et al.*, 2013 ▸), STAR (Bacci *et al.*, 2014 ▸, 2016 ▸), FELICIA (Niknejadi *et al.*, 2019 ▸), ASU-CXLS (Graves *et al.*, 2014 ▸) and Smart-Light (Luiten, 2016 ▸). In the design of these sources, the standard ICS-approach is often considered an intermediate step to super-radiant X-ray sources or compact free-electron-lasers (FELs) by artificial micro-structuring of the electron pulse, which would boost their X-ray flux significantly due to coherent emission of radiation (*e.g.* Nanni *et al.*, 2018 ▸; Fransen *et al.*, 2019 ▸). Storage-ring-based concepts, like ThomX (Variola *et al.*, 2014 ▸), the Tsinghua Thomson X-ray Source-Phase II (TTX-II) (Rui & Huang, 2018 ▸; Liu, 2019 ▸) or the ICS of the Munich Compact Light Source (MuCLS) (Eggl *et al.*, 2016*a* ▸), typically rely on conventional accelerator technology for the same reason. Furthermore, they benefit from the high collision frequency intrinsic to small-diameter storage rings allowing for less demanding constraints on the inter­action geometry for inverse Compton scattering.

In the following, we will focus on the MuCLS facility, which is located at the Munich School of BioEngineering (MSB), Technical University of Munich. It is operated by the Chair of Biomedical Physics in the framework of the Centre for Advanced Laser Applications (CALA) (CALA, 2020 ▸), a joined project of the two Munich universities (Ludwig-Maximilians-University, LMU, and Technical University of Munich, TUM). The MuCLS consists of the inverse Compton source, the so-called Lyncean Compact Light Source, developed and constructed by Lyncean Technologies Inc., and the X-ray beamline with two end-stations developed in-house. First, we briefly review the design of the ICS of the MuCLS. Following this, we describe why – in our opinion – inverse Compton sources are very suited for demanding X-ray imaging applications and which benefits and drawbacks users at ICSs, such as the MuCLS, might experience compared with synchrotron facilities and X-ray tube sources. Finally, we review the different X-ray imaging techniques available at the MuCLS and conclude with an outlook on planned upgrades to the MuCLS.

## 2. Design of the Compact Light Source at the MuCLS   

**Figure 1:**
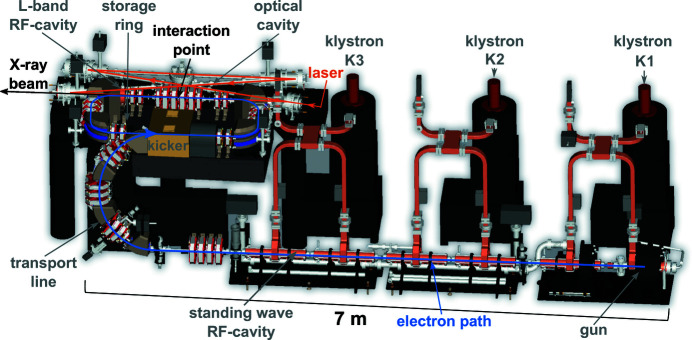
CAD-drawing of the ICS as installed at MuCLS at the Munich School of BioEngineering of the Technical University of Munich in Garching, Germany. The CAD-drawing was provided by Lyncean Technologies Inc. (Fremont, USA).

The fundamental design concept of the ICS of the MuCLS dates back to 1998 when Huang and Ruth proposed the concept of a ‘Laser-Electron Storage Ring’ (Huang & Ruth, 1998 ▸) which was further refined by Loewen (Loewen, 2003 ▸) and Lyncean Technologies Inc. (Fremont, USA) later on. An annotated CAD-rendering of the ICS of the MuCLS is depicted in Fig. 1 ▸. On the lower right side, the gun is visible, where electrons are produced at a copper photo-cathode. The photon energy of the photo-cathode laser has to be higher than the work function of copper (4.6 eV), which is achieved with a frequency-quadrupled Nd:YLF laser system operating at 266 nm wavelength (not shown in the drawing). Its oscillator is operating at 64.91 MHz repetition rate, which corresponds to the revolution frequency of the electrons in the storage ring. A regenerative amplifier is used to pick pulses from this pulse train and amplify them. Frequency-quadrupling is performed by two nonlinear crystals. The UV-pulse is steered to the electron gun, where it produces electron bunches of typically about 250 pC. The charge is kept constant during operation by a feedback adjusting the current through the diode pumping the laser crystal in the regenerative amplifier. Subsequently, these electron bunches are accelerated in up to five S-band (2856 MHz) standing-wave accelerator modules [radio-frequency (RF) cavities] to the desired electron energy between 29 MeV and 45 MeV, which can be freely adjusted choosing the number of accelerating structures and the gradient inside of them adequately. Klystrons, high-power RF-amplifier systems, supply the RF-cavities with the accelerating electric fields. Since the electron energy after acceleration determines the energy of the generated X-ray photons, variation of the X-ray energy between 15 keV and 35 keV is achieved with aforementioned adjustments to the electron accelerator. In the transport line, the semi-circle arc visible below the storage ring on the left of Fig. 1 ▸, the electron bunches are prepared for injection into the storage ring and guided to the fast kicker installed in one of its long straight sections. The latter device kicks the electron bunches onto the storage ring’s orbit. In the opposite long straight section, which is shared with the optical cavity, the installed quadrupole magnets focus the electron bunch to a small size at the interaction point of electrons and laser photons in order to create a high luminescence and recollimate the electrons afterwards. In addition, a short L-band (1428 MHz) RF-cavity is installed right after the quadrupole magnets replenishing electron energy lost by synchrotron radiation and inverse Compton scattering. Exchanging the electron bunch in the storage ring every 40 ms, *i.e.* at a rate of 25 Hz, preserves a circular electron beam shape. In contrast to the photo-cathode laser, the laser used for inverse Compton scattering is based on Nd:YAG technology operating at a wavelength of 1064 nm. This system delivers up to 30 W at a repetition rate of 64.91 MHz. Pulse duration of the sech-shaped laser pulse has been determined with an autocorrelator to be 26 ps and its M^2^-value to be 1.3 in the horizontal as well as vertical direction. Laser pulses delivered by the system are mode-matched to the eigenmode of a high-finesse (>37000) enhancement cavity, a four-mirror bow-tie ring resonator acting as a passive laser amplifier with a gain of >15000. This increases the laser power from ∼ 25 W at the entrance window of the enhancement cavity to ∼ 350 kW. In order to increase luminescence, the cavity also focuses the laser beam in the interaction point, where it counter-propagates to the electron beam. The oscillator of the laser system is kept on resonance with the enhancement cavity via the Pound–Drever–Hall locking scheme (Pound, 1946 ▸; Drever *et al.*, 1983 ▸; Black, 2001 ▸). Coincidence of laser pulse and electron bunch in the interaction point is ensured by locking the round-trip frequency of the cavity to the low-level RF driving electron acceleration. The current parameters of the ICS installed at the MuCLS are summarized in Table 1 ▸.

## 3. Evaluation of the MuCLS in comparison with synchrotrons and advanced X-ray tube sources   

This section compares the MuCLS facility with synchrotrons and other laboratory set-ups based on advanced X-ray tube sources. To this end, the first part of this chapter provides a detailed analysis of these X-ray sources. The parameters of interest are evaluated in the following order: flux, source size, X-ray generation and its effect on X-ray beam divergence and bandwidth. Finally, these parameters are combined into the figure-of-merit for X-ray beams, the brilliance. Subsequently, the three facility types are compared in terms of costs for both installation and operation, support infrastructure and ease of access for users in the second part.

### 3.1. Comparison of the X-ray sources’ beam parameters   

#### 3.1.1. Flux   

**Figure 2:**
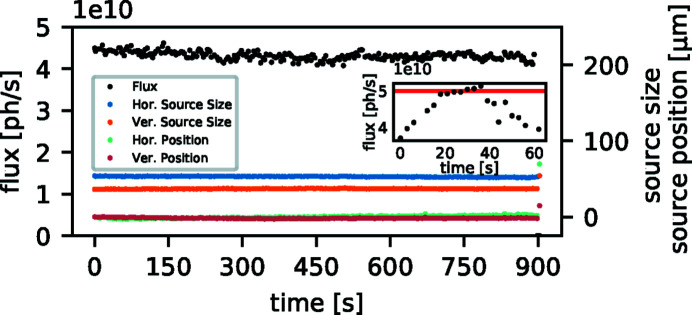
Performance of the MuCLS operated at the 35 keV X-ray configuration. The inset depicts the maximum X-ray flux of 5 × 10^10^ photons s^−1^ achieved under optimal conditions. However, this flux has been reached only briefly during X-ray optimization so far. Typically, small movements of the electron beam within the stability range of the storage ring in conjunction with adjustments of the temporal delay between laser and electron beam are sufficient for daily X-ray tuning. On timescales required for experiments, this X-ray flux stabilizes at a level of 4.0 × 10^10^ to 4.3 × 10^10^ photons s^−1^ under such circumstances, as shown in the main figure. Data were recorded with the beam-monitoring system described by Günther *et al.* (2019 ▸).

Insertion devices at state-of-the-art synchrotrons provide several kilowatts of X-ray power, *e.g.* 9.5 kW at P11 at Petra III (DESY, 2019*a* ▸). This flux enables such sources to deliver up to 1 × 10^13^ photons s^−1^ on the sample (after a double-crystal monochromator). In contrast to this narrow-bandwidth synchrotron beam, the ICS at the MuCLS provides orders of magnitude lower flux within its full bandwidth of 3−5%. From a value of 1 × 10^10^ photons s^−1^ (Eggl *et al.*, 2016*a* ▸) at the time of installation, an upgrade of the laser system increased the flux of the ICS to up to 3 × 10^10^ photons s^−1^ (Günther *et al.*, 2018 ▸) and further optimization of our optical cavity system recently resulted in up to 5 × 10^10^ photons s^−1^ at 35 keV X-ray energy under optimal conditions. A measurement of this peak flux is depicted in the inset of Fig. 2 ▸. However, this peak flux has been reached only briefly during X-ray tuning so far. In such optimal circumstances a stable X-ray flux of 4.0 × 10^10^ photons s^−1^ to 4.3 × 10^10^ photons s^−1^ is achieved on time scales required for experiments, *cf.* the main graph of Fig. 2 ▸. Although micro-focus tubes typically do not reach this X-ray flux level [integrated flux: ∼ 1 × 10^9^ photons s^−1^ (Procop & Hodoroaba, 2008 ▸)], the integrated full-spectrum flux of high-power rotating anodes as well as liquid-metal-jet-based X-ray sources can surpass the one available at the MuCLS. The integral flux of high-power rotating anodes employed in clinical computed tomography (CT) systems is >1 × 10^13^ photons s^−1^ on the detector (Schlomka *et al.*, 2008 ▸). Considering the conversion efficiency from electrical power to X-ray power of ∼10^−4^, the generated X-ray flux is as high as 1 × 10^15^ photons s^−1^ (Behling, 2016 ▸), while liquid-metal jet sources reach >1 × 10^12^ photons s^−1^ to ∼ 1 × 10^13^ photons s^−1^, estimated from the 160 kVp spectrum (Excillum, 2019 ▸).

#### 3.1.2. Source size   

X-ray beams at current third-generation synchrotron facilities have an elliptical source size with their aspect ratio depending on the electron beam β-function at the location of the insertion device. For state-of-the-art synchrotrons, like PETRA III, typical source sizes for a high-β section are 140 µm × 5.6 µm, and 36 µm × 6.1 µm for a low-β one (Barthelmess *et al.*, 2008 ▸). The latter source size is comparable with those of emerging fourth-generation synchrotrons, such as MAX IV [48.7 µm × 6.2 µm (MAX IV Collaboration, 2010 ▸)] and ESRF-EBS [30.2 µm × 5.1 µm (ESRF, 2018 ▸)]. The source size of the MuCLS of radius <50 µm is comparable with synchrotons, at least in the horizontal direction, and larger than the projected X-ray source size of the liquid-metal jet sources of diameter 20 µm (Excillum, 2019 ▸). Microfocus X-ray tubes provide source sizes ranging from a couple of micrometres to a couple of tens of micrometres, with micro-focus rotating-anode sources (70 µm for the Rigaku FR-X) on the large spot size end of the spectrum. Currently, the smallest X-ray source size is provided by nano-focus transmission tubes, such as the Excillum NanoTube N1 60kV which enables spot sizes of 200 nm to 300 nm (Müller *et al.*, 2017 ▸), but their flux is orders of magnitude lower due to the use of thin transmission targets with low heat dissipation capability.

#### 3.1.3. Divergence and bandwidth   

Despite flux and source parameters varying strongly for the different X-ray tube concepts, there is one characteristic which they all have in common: X-ray generation by bremsstrahlung. This results in a wide natural divergence as well as a broad spectrum of X-rays (basically from a few keV to *eU*, where *e* is the electron charge and *U* the acceleration voltage) containing additionally intense, narrow and material-specific emission lines. Therefore, high peak X-ray energies can be reached but high average X-ray energies are feasible only by filtering the spectrum appropriately; however, at the cost of a strongly reduced integral flux.

In contrast to X-ray tubes, ICSs and synchrotron sources use a different mechanism to generate X-rays. X-ray generation at ICSs can be described equivalently to an undulator considering the electro-magnetic field of the laser as a ‘laser-undulator’. Consequently, the divergence of their X-ray beam is inversely proportional to the γ-factor, *i.e.* electron energy, which is typically in the range of a few tens of MeV. The resulting divergence is a few milliradians to a few tens of milliradians, 4 mrad at the MuCLS defined by a fixed aperture. In contrast, the beam divergence is in the range of a few microradians to a few tens of microradians for synchrotrons due to their electron energies of a few GeV. Contrary to this disparity in divergence between ICSs and synchrotron sources, their spectra can be very much alike, *i.e.* with a natural bandwidth of a few percent. However, due to the significantly reduced flux at ICSs compared with synchrotrons, the former seldom use monochromators to further filter the spectrum, which could reduce their bandwidth down to Δ*E*/*E* ≃ 10^−4^ with a double-crystal monochromator.

#### 3.1.4. Brilliance   

A figure-of-merit combining the aforementioned X-ray source parameters is the brilliance. For synchrotrons, the corresponding brilliance is in the range of 1 × 10^20^ to 1 × 10^21^ photons  s^−1^ mrad^−2^ mm^−2^  (0.1% bandwidth)^−1^ (PETRA III, Hamburg, Germany) (DESY, 2019*b* ▸), which is many orders of magnitude higher than for rotating anodes [up to 1.2 × 10^10^ photons s^−1^ mrad^−2^ mm^−2^ (Skarzynski, 2013 ▸)]. For 70 kVp and 200 W electron beam energy, liquid-metal jet sources reach a value of 5 × 10^10^ photons s^−1^ mrad^−2^ mm^−2^ (0.1% bandwidth)^−1^ at their Ga *K*_α_ line, where these sources are most brilliant, while their brilliance is ∼ 2 × 10^9^ photons s^−1^ mrad^−2^ mm^−2^ (0.1% bandwidth)^−1^ for the In *K*_α_ line (Wansleben *et al.*, 2019 ▸). It has to be considered that the natural line-width of 2.6 eV for gallium and 10.6 eV for indium was scaled to the 0.1% bandwidth level of 9.25 eV and 24.4 eV in this calculation, respectively. Furthermore, Wansleben *et al.* (2019 ▸) collimated the X-ray beam to 2.5 × 10^−5^ sterad. This might lead to a slight overestimation of the spatially averaged brilliance, as self-absorption for off-axis angles of the beam with a full opening angle of 30° (0.21 sterad) is not taken into account. However, considering the radiant flux at the Ga *K*_α_ line of 6 × 10^12^ photons s^−1^ sterad^−1^ [4.6 × 10^6^ photons s^−1^ mrad^−2^, when expressed in terms of beam divergence] reported in their work, the total X-ray flux emitted from this line into the maximum full angle of 30° is ∼ 1.3 × 10^12^ photons s^−1^. The ICS of the MuCLS is similarly brilliant to liquid-metal jets [1.2 × 10^10^ photons s^−1^ mrad^−2^ mm^−2^ (0.1% bandwidth)^−1^]. Yet, it has the advantage that its brilliance is almost constant over the whole energy range. In contrast, the brilliance of bremsstrahlung-based sources drops by several orders of magnitude at energies other than the characteristic emission lines. Responsible for this behaviour at the MuCLS is the interaction cross-section of inverse Compton scattering. For our X-ray energy range, it can be approximated by the Thomson cross-section and therefore as being energy independent. Note that the increase in total flux observed at the MuCLS when increasing X-ray energy is due to the decrease of the natural emission angle (∼1/γ) when increasing the electron energy. As a consequence, a larger portion of the total flux is extracted through the fixed X-ray exit aperture and the spectrum is broadened slightly, while the intensity at the peak stays constant.

### 3.2. Discussion of typical facility implementation of aforementioned X-ray sources   

In the discussion so far, only machine parameters have been discussed, although these parameters do not provide an exhaustive evaluation of the X-ray source most suitable for specific tasks / a specific research environment. First of all, the instrument itself has to be acquired, maintained and operated. X-ray tube-based systems are inexpensive compared with other types of X-ray sources, turn-key, fit into a small laboratory and require little maintenance. Thus such an instrument may also be afforded and operated by a small research group, with the option of daily use. Contrary to that, ICS facilities like the MuCLS require a multi-million Euro commitment, a larger laboratory only for the X-ray source itself, elaborate radiation shielding due to the higher electron energies and trained operators for operation and maintenance. All of this significantly increases the costs for acquisition and operation compared with X-ray tube-based systems. Consequently, such X-ray sources will be mainly incorporated into central research or service facilities of bigger universities or larger non-university research institutes.

In contrast to X-ray tube-based set-ups which are usually optimized for a specific task (*e.g.* crystallography or microtomography), this also means that experimental end-stations in such an environment have to be more flexible and have to provide complementary techniques in order to exploit the full potential of these sources. Furthermore, integration into a central facility also means that a better support infrastructure is going to be available at an ICS to satisfy requirements of the members of such an institution, like various sample preparation laboratories or complementary instruments. At the moment, the support infrastructure for the MuCLS available at CALA and the Munich School of BioEngineering include dedicated chemistry and electronic laboratories, different micro-CT systems, a clinical CT scanner as well as different light and electron microscopes, thereby surpassing the usual instrumentation of an average research group. While such support infrastructure might cover the main applications of the regular (internal) facility users, infrastructure for exotic experiments or external users might be missing. Such support infrastructure might be available at synchrotrons which provide a wide range of central laboratories for sample preparation or additional characterization, which becomes feasible since they serve many beamlines at the same time. Moreover, synchrotron facilities employ a lot of staff for facility operation and user support at the beamline during experiments.

**Figure 3:**
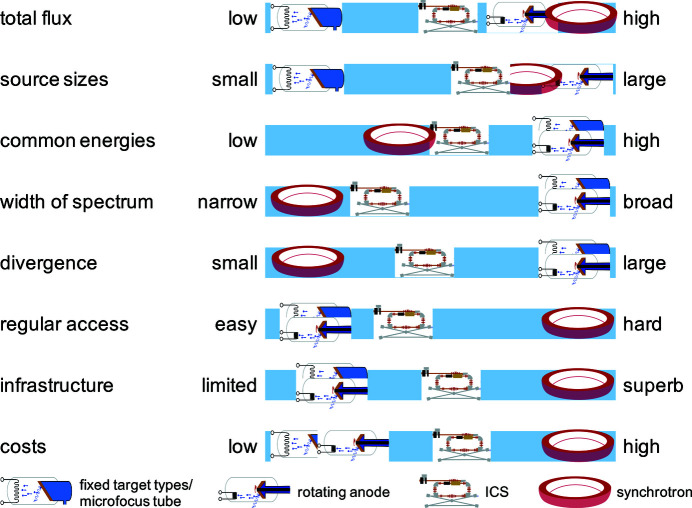
Typical X-ray source parameters, available support infrastructure and system costs (operation as well as acquisition/development) are depicted qualitatively for synchrotrons, ICS and X-ray tubes (subdivided into micro-focus tubes and rotating anodes). The combination of synchrotron-like radiation, especially in terms of energy range, spectral bandwidth and X-ray beam divergence, with easy access is unique to inverse Compton sources. Therefore, applications at ICSs should rely on them ideally.

Although synchrotrons provide the highest brilliance and probably best infrastructure, which might be optimal for the experiment, they have some drawbacks. First of all, getting beam time at synchrotrons is often difficult since most beamlines are heavily oversubscribed. As a consequence, if granted, time for experiments is very limited. Furthermore, samples have to be transported to the facility, which can be complicated for certain classes of samples due to transport or import restrictions. Moreover, transportation could lead to degradation or damage of sensitive specimens, or requires them to be prepared on-site in an unfamiliar laboratory. In contrast, a central facility can offer easy regular on-site access to all members of the host institution. This also makes it easier to handle samples which are tricky to transport, either because they are too delicate or due to restrictions. In particular, it allows the on-site users to prepare the samples in their own laboratories and even take them back there for further treatment steps between parts of the X-ray experiments. The MuCLS facility was established as part of CALA with these advantages in mind. Access to CALA facilities is open to members of the two universities, TUM and LMU, and their collaborators. At the moment, there is thus no proposal system, but access to external users is granted through collaborations with local groups instead. External users who are interested in establishing a collaboration to perform experiments at the MuCLS are asked to use the contact information on http://www.bioengineering.tum.de/en/central-building/munich-compact-light-source/ for inquiries. The preceding discussion is summarized in Fig. 3 ▸, which provides a qualitative overview of the aforementioned parameters for the different X-ray sources.

One technique that is well suited to the MuCLS’s source properties is dynamic (high-resolution) phase-contrast X-ray imaging. The larger (and symmetric) cone angle compared with synchrotrons enables larger objects, like the lungs of mice, to be captured in a single exposure with shorter acquisition times than other laboratory X-ray sources [except for liquid-metal jet ones at much lower resolution, *cf*. Preissner *et al.* (2018 ▸)], which in turn enables *in vivo* small-animal phase-contrast imaging. Consequently, X-ray imaging so far seemed to be the most suited candidate for transferring techniques limited to synchrotrons into a laboratory. Thus we designed flexible experimental end-stations keeping this goal in mind, which enables us to perform (propagation-based phase-contrast) microtomography, dynamic *K*-edge subtraction imaging, grating-based phase-contrast imaging and propagation-based imaging.

## 4. The MuCLS beamline   

**Figure 4:**
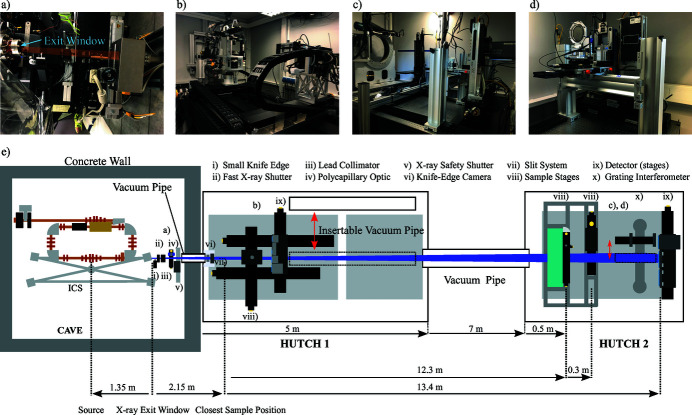
The MuCLS X-ray beamline. (*e*) Schematic drawing (not to scale) of the MuCLS consisting of the ICS housed in a radiation shielding enclosure, the beamline front-end located right after the exit window of the ICS at a distance of 1.35 m to the interaction point and the two end-stations designed for complementary X-ray imaging methods. The shortest source-to-sample distance is 3.0 m and the longest one is 15.6 m. (*a*) Photograph of the front-end inside the radiation protection enclosure with the X-ray exit window on the left side. (*b*) Possible experimental set-up in hutch 1 including a polycapillary optic and silicon drift detector. (*c*) The whole set-up of end-station 2. (*d*) Photograph of the grating interferometer and the installed detectors.

The versatile X-ray application beamline of the MuCLS has been designed to accommodate a variety of imaging techniques as well as enabling the implementation of other techniques, if desired. A schematic to-scale floor plan of the whole MuCLS facility is shown in Appendix *A* [App appa]. X-rays exit the ICS of the MuCLS at a distance of 1.35 m to the X-ray source point at a height of 1.67 m above ground with a divergence of 4 mrad. This exit window defines the starting point of the MuCLS beamline, which is schematically depicted in Fig. 4 ▸ (bottom row) together with photographs of selected parts (Fig. 4 ▸, top row). It can be divided into three sections: a front-end section and the two experimental end-stations. The beamline control system is *SPEC* (Certified Scientific Software, Cambridge, USA) which was chosen because of its built-in hardware support, its flexibility to add own code through user macros and its widespread use at many synchrotrons. This makes it easy for users to integrate their own equipment, if required, or existing *SPEC* routines which have been already developed for measurements at other facilities. At the MuCLS, the built-in hardware support directly interfaces with the motor controllers. Custom macros control the detectors through TCP/IP connections in a client–server model. Specifically developed data acquisition macros are optimized to automate common measurement tasks (*e.g.* CT scans) while providing the necessary metadata for the in-house data processing pipeline.

### 4.1. The MuCLS front-end   

The front-end [Fig. 4 ▸(*a*)] contains a fast X-ray shutter (model SH-10-PI-B-L-24; Electro-Optical Products Corp., Ridgewood, USA), a knife-edge combined with a special camera system for X-ray beam diagnostic and closed-loop X-ray beam stabilization (Günther *et al.*, 2019 ▸), a polycapillary optic (IFG, Berlin, Germany) with a long working distance and thus mildly focusing the beam to a diameter of ∼ 3 mm at the sample position in the first end-station, the X-ray safety shutter (in-house design, motorized translation from Festo, Esslingen, Germany) and a slit system (Huber Diffraktionstechnik, Rimsting, Germany) at the very end for beam shaping. The fast X-ray shutter can be controlled by *SPEC* and can be used to reduce the dose applied to the specimen under investigation, when synchronized with data acquisition. The polycapillary optic located in the front-end has originally been designed for micro-beam radiation therapy studies enabling the implementation of this technique in the first experimental end-station. This is much more convenient than performing experiments inside the ICS enclosure like in the first trials (Burger *et al.*, 2017 ▸). The recently developed custom-built X-ray beam monitor records the X-ray source parameters (flux, source size and -position) continuously. This enables manual re-optimization of the collision of the laser and electron beams even during experiments. Moreover, data provided by this device can be used for a closed-loop feedback system stabilizing the X-ray position significantly. Details on the system and its performance can be found in the publication by Günther *et al.* (2019 ▸). In addition, drops in X-ray flux, typically related to a loss of lock of the laser system to the enhancement cavity, can be detected *in situ* and used to automatically pause experiments thereby reducing data loss.

### 4.2. MuCLS end-station 1   

The first experimental end-station houses two optical tables for experiments. The first one is equipped with a very flexible multi-purpose set-up depicted in Fig. 4 ▸(*b*), which has been employed for microtomography, propagation-based phase-contrast imaging and X-ray absorption spectroscopy so far. The sample manipulation system, as well as the detector assembly, are mounted on two different motorized stages (Limes 170; Owis, Staufen, Germany) with 1.5 m travel range along the X-ray beam axis each. Both systems can be completely moved out of the beam sideways by two additional motorized linear stages, which is necessary to perform experiments in the second end-station. The shortest source-to-sample distance achievable is 3.5 m. Therefore the maximum source-to-sample distance is 5 m, whereas the maximum sample-to-detector distance is ∼ 1.8 m. Accordingly, the X-ray beam diameter ranges from 14 mm to 20 mm. The sample is mounted on a rotation stage (either a PI Micos PRS-200 or a LAB RT-150U) placed on top of an elevator stage (Huber Diffraktionstechnik, Rimsting, Germany) with 90 mm range. Specimens can be centred on the rotation axis with two Newport MFA-PPD stages on which the actual sample holder (*e.g.* a goniometer head) can be mounted. To further enhance the set-up’s flexibility, the rotation stage is located inside a custom-built housing which is covered by a small breadboard (22.5 cm × 33 cm) with a threaded hole pattern (M6 on 25 mm square grid). It also contains an opening with 100 mm diameter around the centre of the rotation axis. This breadboard can thus be used to mount additional equipment, *e.g.* a focusing optic with a short working distance, closely to the specimen while the latter can be rotated freely, nevertheless. A multi-axis overhead sample manipulation system can be mounted onto this small table if two axes of rotation or similar are required. The X-ray detectors are mounted on a horizontal breadboard on top of an elevator stage (Humes 200; Owis, Staufen, Germany) to centre each detector in the X-ray beam. At the moment, mainly cameras with pixel sizes around 10 µm and fibre-optically coupled scintillators are installed, but a system with 4× or 10× optical magnification is available, too. Details on the detectors are summarized in Table 2 ▸. Furthermore, a Ketek AXAS-D silicon-drift detector (Ketek GmbH, Munich, Germany) is available, *e.g.* for measurements of the source spectrum or fluorescence. For the latter, a second polycapillary optic (IFG, Berlin, Germany) can be installed, which focuses the X-rays to a ∼ 50 µm spot at a working distance of 17 mm. The second optical table is currently reserved for temporary set-ups, *e.g.* of users who want to use their own equipment.

### 4.3. MuCLS end-station 2   

The second end-station is installed at a distance of 14.8 m to the source point and contains two permanently installed sample manipulation systems, an optional two-grating interferometer for differential phase-contrast imaging (DPC) and detectors with pixel sizes in the range from 74.8 µm to 172 µm, which cover the full field of view of ∼ 60 mm in diameter. Details about the available detector systems (source-to-detector distance ∼ 16.4 m) are presented in Table 3 ▸.

Three of these detectors can be installed at the same time. They are centred in the beam with two stages for horizontal and vertical movement. Detectors and interferometer are installed on an optical table with passive air damping. This avoids the problem of external vibrations, as well as those arising from specimen movement with the sample manipulation stages, being coupled into the interferometer, which would strongly deteriorate its performance. The interferometer [Fig. 4 ▸(*d*)] consists of two gratings with an effective area of ∼65 mm in diameter covering the full beam. Currently, two different nickel phase gratings (G1) are available for design energies of 25 keV and 35 keV. In order to facilitate the lithographic production process, the same periodicity *p* (4.92 µm) was chosen for both phase gratings, and a single gold absorption grating (periodicity 5.0 µm) was manufactured by the Karlsruhe Nano Micro Facility (KNMF, Eggenstein-Leopoldshafen, Germany). Thus, the phase grating for 35 keV has to be tilted by 6.3° to generate the design periodicity of 4.89 µm. Grating properties are summarized in Table 4 ▸. In grating-based phase-contrast imaging, one of the two gratings is stepped over one period. This phase stepping generates a sinusoidal intensity curve for each pixel, whose modulation strength, the visibility, is a crucial factor for the sensitivity of the interferometer. At 25 keV a visibility of >45% can be achieved while those at 35 keV is >30%. DPC at X-ray energies in between is possible by choosing the G1, whose design energy is closer to the desired energy, and tilting it until the desired periodicity is reached. The latter can be calculated by (Jud, 2019 ▸)
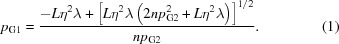
For the available G1s (phase-shift of π/2), η = 1 and the source-to-interferometer distance *L* ≃ 16 m. *n* denotes the Talbot order (the current design is *n* = 1), and λ the X-ray’s wavelength with an energy *E*. The corresponding inter-grating distance *d* can be approximated by (Jud, 2019 ▸)
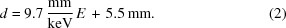
Consequently, the grating holders are mounted on a sturdy rail running along the beam axis as the inter-grating distance has to be adjusted for different energies. The rail itself is mounted on a second one running perpendicular to the beam axis which allows for sliding the whole interferometer out of the beam without impairing its alignment. The end-station has two sample manipulation systems. They are mounted on an extra support frame. This decoupling prevents negative effects of sample stage movements on the stability of the grating interferometer. One system is built for projection imaging of large specimens, *e.g.* pig hearts for angiography, or mastectomy specimens. Therefore this system has a breadboard as sample support which can be translated by two linear stages (LTM 120; Owis, Staufen, Germany) in the plane perpendicular to the X-ray beam axis. The key component of the second system, depicted in Fig. 4 ▸(*c*), is a large stage for rotation of the sample around the X-ray beam propagation axis (Goniometer 440; Huber Diffraktionstechnik, Rimsting, Germany). In combination with the grating interferometer, it enables directional dark-field imaging, so-called X-ray vector radiography (XVR) (Jensen *et al.*, 2010*b* ▸). Two linear stages (LTM 80; Owis, Staufen, Germany) are attached to this rotation stage for translation of the sample perpendicular to the beam axis. The specimen itself is mounted onto a second rotation stage (DMT 65; Owis, Staufen, Germany) used for tomography and placed on top of the translation stages. Consequently, this end-station is chosen if DPC or XVR measurements are performed or large samples are imaged.

## 5. X-ray imaging at the MuCLS   

In the following, we give a brief overview on the different X-ray imaging techniques that are currently available at the MuCLS and present some application examples.

### 5.1. X-ray microtomography   

**Figure 5:**
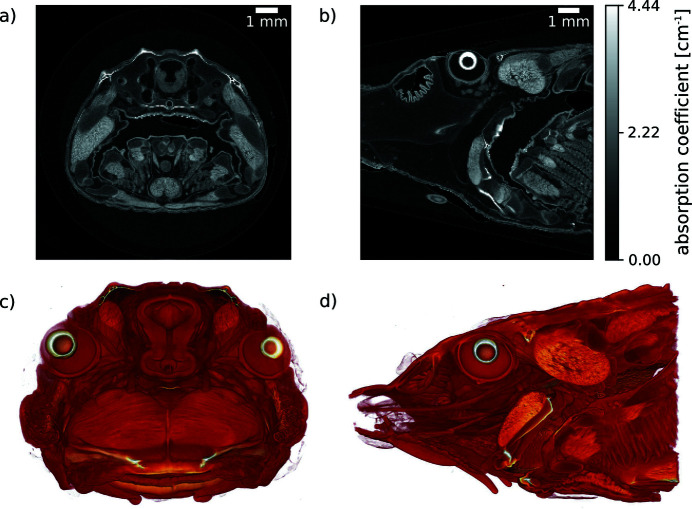
Micro-CT (2500 projections over 360°, exposure time 0.4 s per projection) of a sturgeon fish head acquired at an X-ray energy of 25 keV with the Ximea detector (9 µm pixel size). (*a*, *b*) Axial and sagittal slices reconstructed applying filtered back-projection. (*c*, *d*) Volume renderings with direction of view corresponding to (*a*) and (*b*).

With the goal of combining the benefits of the ICSs of the MuCLS with fast dynamic imaging, our high-resolution imaging and microtomography (micro-CT) efforts have been focusing on the ∼10 µm resolution range. For this, we use very efficient detector systems based on fibre-coupled scintillators instead of optical coupling. Since the X-ray beam divergence is 4 mrad, geometric magnification is very low. On the one hand, this inhibits implementation of projection microscopy for this resolution range as often used in cone-beam micro-CT systems with detectors with large pixels, *e.g.* hybrid pixel array detectors. On the other hand, a low-divergence beam allows for long source-to-sample distances. This increases the coherence length without impairing flux density on the sample and is thus beneficial for phase-contrast imaging. Phase imaging techniques are discussed in more detail in Sections 5.3 and 5.4. As a result, X-ray microtomography at the MuCLS mainly benefits from the increased sensitivity (Töpperwien *et al.*, 2018 ▸) and flux density, while in addition the X-ray source’s quasi-monochromatic spectrum prevents beam-hardening artefacts (cupping) in CT (Achterhold *et al.*, 2013 ▸). An example where the latter benefits come into play is presented in Fig. 5 ▸ depicting a sturgeon fish head, which was treated with a molybdenum-based stain (Handschuh *et al.*, 2017 ▸) before the micro-CT measurement. At an X-ray energy of 25 keV, 2500 projections, each with an exposure time of 0.4 s, were acquired over 360° with the Ximea camera. An axial and a sagittal slice of the volume reconstructed with filtered back-projection are shown in Figs. 5 ▸(*a*) and 5(*b*), respectively. Figs. 5 ▸(*c*) and 5(*d*) are volume renderings with directions of view corresponding to Figs. 5 ▸(*a*) and 5(*b*). The absence of beam-hardening artefacts can be inferred from Fig. 5 ▸(*a*). Moreover, the features of interest of the inner fish anatomy are resolved very well at high contrast, *cf*. Figs. 5 ▸(*a*) and 5(*b*), which provides an example for the excellent capabilities of our microtomography system for applications which require a resolution of ∼ 10µm.

Notwithstanding the MuCLS’s great performance in microtomography, two properties of the X-rays produced at ICSs make high-resolution microscopy, which relies either on coherent X-rays, like coherent diffractive imaging and ptychography, or on focusing optics [scanning transmission X-ray microscope, projection microscope and full-field transmission X-ray microscope (TXM)], not that straightforward. First, X-ray flux at ICSs is significantly lower than at synchrotrons, which requires very efficient optics in order to generate high-resolution images at short acquisition times. Second, the divergence angle of X-rays produced at ICSs is orders of magnitude larger (mrad instead of µrad). This requires the optics to be placed either very close to the X-ray source or to be quite large in order to intercept the whole beam. In the case of the MuCLS, this is complicated by the fact that the ICS there is based on a storage ring for the electron beam and an enhancement cavity for the laser. Accordingly, no X-ray optic may be placed inside the laser cavity, limiting the minimum distance of the optic to the X-ray source to ∼1.4 m, where the beam diameter is already nearly 6 mm. This is far too large for Fresnel zone-plates and typical compound refractive lenses. Lyncean Technology Inc. has developed a multilayer-based KB-mirror system, which allows 1:1 refocusing and in principle could be used as, for example, a condensor for a TXM or for crystallography. However, if the distance between the machine and the wall of the radiation projection enclosure is increased, the closest distance at which the unfocused beam can be used also becomes larger, which results in a – potentially undesirable – larger beam diameter. To circumvent this problem, Lyncean’s current design places the optics into the radiation protection enclosure wall itself at a source-to-mirror distance of ∼ 2 m. The required large hole in the shielding wall raised radiation protection concerns at our institute, whereupon this system was not acquired together with the source. Without a condensor optic, image acquisition at an X-ray energy of 25 keV took at least 300 s with a Rigaku XSight Micron LC CCD-camera set to an effective pixel size of 0.54 µm in order to obtain an acceptable signal-to-noise ratio. However, considering the current X-ray flux after several upgrades, the acquisition time should be lower by a factor of three at least. Nevertheless, optical magnification significantly narrows down the field of view, in this case to 1.80 mm × 1.36 mm, whereas the X-ray beam itself is 18 mm in diameter. A maximum flux of 3 × 10^10^ photons s^−1^ at 25 keV corresponds to a flux density of ∼ 118  photons s^−1^ µm^−1^ on the detector which covers only 0.96% of the X-ray beam at this distance. Based on these considerations, high-resolution microscopy with either X-ray optics and/or magnifying visible-light optics has not been implemented as a standard technique at the MuCLS for now.

### 5.2. Contrast enhanced and *K*-edge subtraction imaging   

Monochromatic or quasi-monochromatic spectra with tunable X-ray energy provide means to increase the signal-to-noise ratio compared with bremsstrahlung spectra in contrast enhanced imaging, *e.g.* in coronary angiography, where iodine contrast agent is necessary to visualize the arteries. The reason for this is that the spectrum of the MuCLS can be adjusted to lie directly above the *K*-edge of iodine resulting in a higher average absorption coefficient for the MuCLS spectrum than the average one for a bremsstrahlung spectrum. Consequently, the contrast between the iodine-containing parts and surrounding tissue was increased by 20% in an experiment with a porcine heart *ex vivo* (Eggl *et al.*, 2017*a* ▸,*b* ▸).

**Figure 6:**
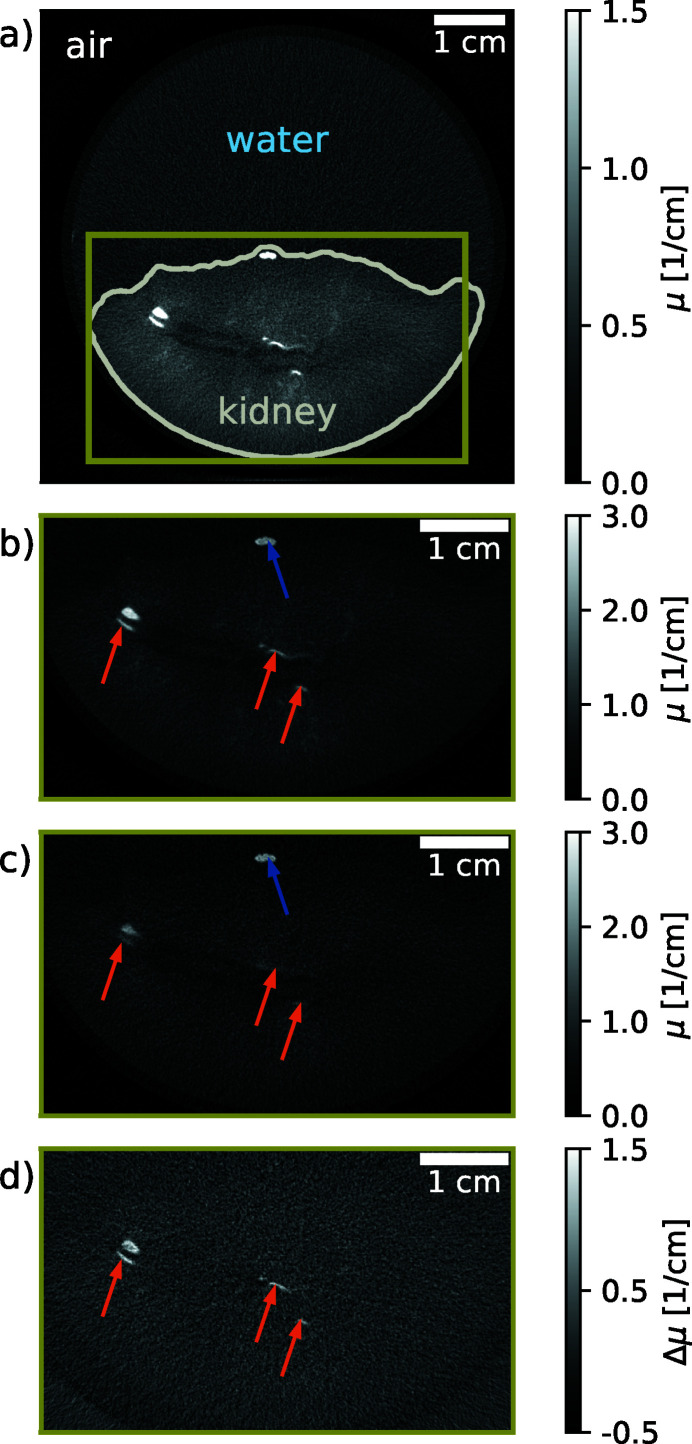
*K*-edge subtraction imaging. (*a*) Full slice of a porcine kidney containing one kidney stone where an iodine solution (Imeron; Bracco Imaging Deutschland GmbH, Germany; 400 mg ml^−1^) was injected into the arteries. The kidney was placed into a plastic beaker glass filled with water. The dynamic range of the image has been adapted for water and soft tissue and the contour of the kidney is plotted in grey for better visibility. (*b*) The same slice cropped to the region indicated in green in (*a*) which contains the kidney. Here and in the following sub-figures the full dynamic range is displayed. (*c*) Image of the same sample recorded with an iodine filter. (*d*) The same slice of the resulting *K*-edge subtraction volume containing only the iodine, *i.e.* the material with the *K*-edge. Orange arrows point to vessels containing iodine contrast agent, while the blue arrow indicates the kidney stone.

Another, more sophisticated, technique, that exploits the step-increase in absorption resulting from a material’s dependent energy of the *K*-shell transition, is *K*-edge substraction (KES) imaging (Rubenstein *et al.*, 1981 ▸). Image contrast for a specific material can be generated by subtracting two images from each other, one taken at an energy just above this material’s *K*-edge and the second one at an energy just below. As the materials absorption coefficient is a continuous function of energy and therefore barely changes between the two images for all materials except the one with a *K*-edge, only the material of interest remains visible in the subtraction image. Although this technique is very useful, its efficient application in the laboratory is difficult when using X-ray tubes, as the required narrow bandwidth spectra can be generated with Ross-filter-pairs only (Arhatari *et al.*, 2017 ▸). This results in very long data acquisition times, because of the tubes’ inherently low brilliance away from their characteristic emission lines. Consequently, this technique has been mainly performed at synchrotrons. However, the MuCLS’s brilliance is orders of magnitude higher than that for the bremsstrahlung continuum of X-ray tubes, which makes *K*-edge subtraction imaging compatible with standard diagnostic imaging in a laboratory. In order to switch the mean energy of the X-rays without changing the ICS’s energy configuration, we used an iodine filter, following the approach of Umetani *et al.* (1993 ▸). Being able to perform *K*-edge subtraction imaging with an ICS that could in principle fit into a clinical environment is an important perspective for clinical diagnostic imaging in which contrast agents are often employed to increase soft-tissue or blood to soft-tissue contrast. An example is the separation of iodine and calcium which is important, for example, in the detection of atherosclerosis. In a test experiment for angiography, we demonstrated the separation of iodine, which was injected as contrast agent into the arteries, and bone. This enabled the observation of vessels behind bones, which otherwise would have been oblique (Kulpe *et al.*, 2018 ▸). Fig. 6 ▸ shows an example for the separation of iodine from tissue and calcium. A porcine kidney with a human kidney stone placed onto its surface (as it did not contain any kidney stones naturally) was chosen for this purpose. In addition, an iodine solution (Imeron; Bracco Imaging Deutschland GmbH, Germany; 400 mg ml^−1^) was injected into the arteries and the ICS of the MuCLS was set to a peak energy of 33.7 keV. 1000 projections were taken over 360° for each of the two tomographies. The acquisition time for a single frame was 44 ms. Fig. 6(*a*) ▸ displays a full reconstructed slice of the unfiltered tomography. Fig. 6(*b*) ▸ is the same slice cropped to the region containing the kidney, and Fig. 6(*c*) ▸ is the same region of the same slice for the filtered tomography. A flat-panel detector (Dexela 1512; Perkin Elmer Inc., USA) with a Gd_2_O_2_S:Tb scintillator was used for image acquisition, and provided an effective pixel size of 70 µm for the projection images. A dark current as well as reference correction of the projections was performed first. The centre shift and tilt of the rotation axis were determined and corrected in a second step. Computed tomograms were reconstructed from these projections by a statistical iterative reconstruction algorithm (Fessler, 2000 ▸) employing a Huber penalty (Huber, 2011 ▸) in the regularization with a strength of 10^−10^ and 15 iterations. In Fig. 6 ▸, the reconstructed slices are cropped to the region containing the kidney. Image reconstruction followed the filter-based approach for KES imaging in Kulpe *et al.* (2018 ▸), which is very well suited to the spectral bandwidth available at the MuCLS. It has been extended to CT (Kulpe *et al.*, 2019 ▸) as well as time-sequence imaging (Kulpe *et al.*, 2020 ▸). In the KES-image, Fig. 6(*d*) ▸, no calcium contribution is visible which enables the separation of vessels containing contrast agent from kidney stones. Combining information from the three images allows to discriminate materials with similar absorption but different chemical composition, as demonstrated here for the bio­medical problem of identifying kidney stones in vessels laced with contrast agent for better visibility. Although all of the mentioned applications have been performed at the iodine *K*-edge, this technique can be applied to all specimens containing elements whose *K*-edge is covered by the energy range that is provided by the ICS of the MuCLS [*cf*. Fig. 11(*c*)].

KES imaging with an ICS might also be an interesting alternative in contrast-enhanced spectral mammography. Consequently, a research programme was initiated to evaluate its potential and performance compared with other techniques, like material decomposition and clinically employed polychromatic contrast-enhanced mammography (Heck *et al.*, 2019 ▸).

### 5.3. Propagation-based phase-contrast imaging   

**Figure 7:**
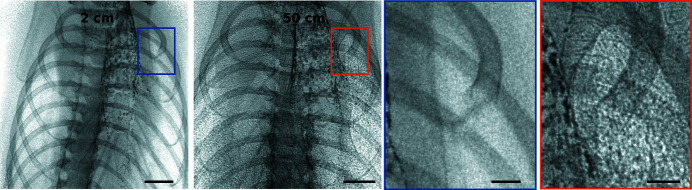
Effect of increasing the sample-to-detector distance from 2 cm to 50 cm when imaging the respiratory system of a mouse. The magnified region clearly shows the enhanced contrast at the edges of the lung. Inside the lung, a speckle pattern is seen which arises from the scattering from the small air sacs in the lung (alveoli). These chest images show that the intensity projection image (edge-enhanced image), recorded at a certain sample-to-detector distance, contains a mixture of contributions from both the absorption (*e.g.* the contrast of the bones) and the phase shifts (*e.g.* the contrast of the lung tissue) in the sample. This means that, in general, the edge-enhanced images only deliver qualitative data. Detector: Hamamatsu, 6.5 µm pixel size, 0.5 s exposure time. Scale bars: 2 mm, magnified area: 0.5 mm. 25 keV X-rays, flux 1.7 × 10^10^ photons s^−1^. Flat-field and dark-current corrected. Linear grey-scale.

In general, the change in the real part of the refractive index is significantly stronger than the one in the imaginary part for weakly absorbing materials in the X-ray regime. Consequently, their image contrast is improved when applying phase-contrast techniques. In terms of instrumentation, the simplest approach for X-ray phase-contrast imaging is propagation-based phase-contrast imaging (PBI). It relies on interference by free-space propagation of a (partially) coherent beam in the Fresnel regime, which translates the objects phase shift into a measurable intensity modulation (Snigirev *et al.*, 1995 ▸; Cloetens *et al.*, 1996 ▸), see Fig. 7 ▸. This intensity modulation is proportional to the Laplacian of the phase shift. As a consequence, step transitions in the refractive index are pronounced, for example at air–tissue interfaces of the lung. Therefore, this observed effect is also called edge enhancement.

**Figure 8:**
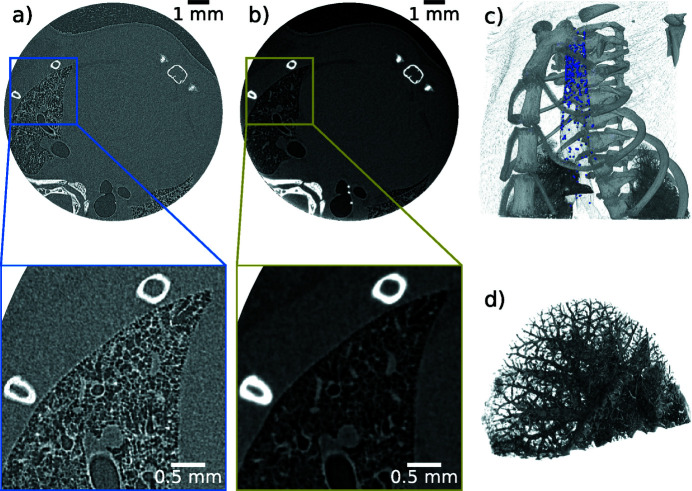
Fast tomography of the chest region of an *ex vivo* mouse, displayed with (*a*) edge-enhancement and (*b*) the result obtained using phase retrieval with the Paganin algorithm with a δ-to-β ratio optimized for lung soft tissue [thus bony structures are blurred (*e.g.* Beltran *et al.*, 2010 ▸)]. (*c*) Combined rendering of the data-set. Only the lungs are rendered using the phase-retrieved reconstruction, the other parts are from the edge-enhanced reconstruction. The high-refractive-index glass beads in the respiratory system are shown in blue. (*d*) Segmentation and rendering of the upper part of the left lung lobe of the phase-retrieved reconstruction. Note that 2 × 2 binning was used, resulting in an effective pixel size of 13 µm of the Hamamatsu detector. The sample-to-detector distance was 0.5 m. 1500 projections were acquired over 360° in 5 min while rotating continuously at a speed of 1.2° s^−1^ with 0.2 s × exposure time per projection. The input value for phase retrieval was δ/β = 1891, an approximation for lung tissue described by H_10_C_0.83_O_5_ (Mohammadi *et al.*, 2014 ▸).

**Figure 9:**
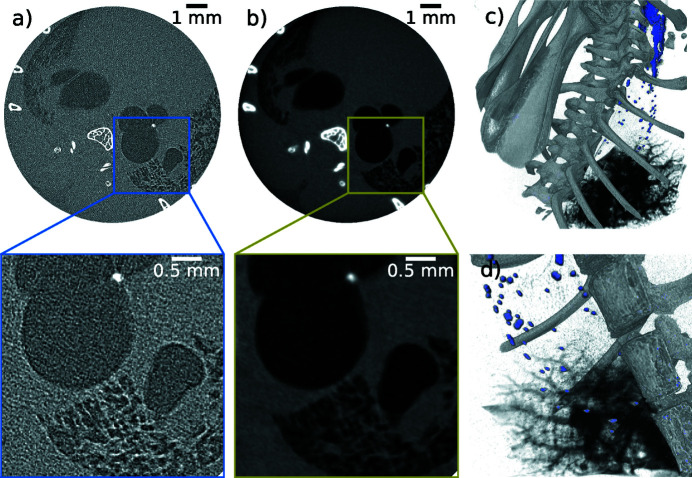
Fast tomography of the chest region of an *in vivo* mouse, displayed with (*a*) edge-enhancement and (*b*) the respective result from the Paganin reconstruction with a δ-to-β ratio optimized for lung soft tissue. (*c*) Combined rendering of the data set. One side of the lung is segmented and rendered using the phase-retrieved reconstruction; the bones and beads are from the edge-enhanced reconstruction. The high-refractive-index glass beads in the respiratory system are shown in blue. (*d*) Zoomed-in region, showing that even in an *in vivo* mouse small airways remain visible, albeit with some motion blur. 2 × 2 binning is used for reconstruction, resulting in an effective pixel size of 13 µm of the Hamamatsu detector. 1000 frames were acquired over 360° at an acquisition time of 0.2 s. Since the images had to be taken during a short breath hold, the rotation speed was reduced to 0.4° s^−1^ resulting in a total time of 15 min.

After PBI with an ICS had been demonstrated first by Ikeura-Sekiguchi *et al.* in 2008 (Ikeura-Sekiguchi *et al.*, 2008 ▸), we could show at the MuCLS that the coherence provided by the ICS is sufficient for quantitative PBI at short acquisition times using a phantom of a single material (Gradl *et al.*, 2017 ▸). Moreover, PBI at the MuCLS can be advantageous compared with liquid-metal jet sources, as demonstrated by Töpperwien *et al.* (2018 ▸). Once the feasibility of this method at the MuCLS was demonstrated, our efforts have been focused on enabling dynamic phase-contrast imaging in a laboratory environment. A very important application which requires dynamic imaging is pre-clinical research on the progression of diseases and response to drug treatment. For these scientific questions, longitudinal studies are indispensable, so regular beam time over an extended period of time is required, which is difficult or impossible to realize at standard synchrotrons. As PBI allows for single-shot data acquisition, it is extremely well suited for non-repetitive motion and time-sequence imaging. Considering the X-ray energy range at the MuCLS, the beam diameter and the corresponding flux density at the first experimental end-station, a focus has been on dynamical studies of small animal models *in vivo*. More specifically, we tried to assess indicators for the health of the respiratory system as a first prerequisite for successful longitudinal studies using this dynamical information. In this context, the velocity distribution of particles injected into the trachea has been identified as such an indicator in synchrotron experiments (*e.g.* Donnelley *et al.*, 2014 ▸), as the mucociliary transport or clearance of particles from the airways is impaired in some conditions, such as cystic fibrosis (Knowles & Boucher, 2002 ▸). We could show that this type of experiment can also be carried out at the MuCLS (Gradl *et al.*, 2018 ▸). This can be used to track disease development or treatment response which is foreseen to be an important application in the future. In order to investigate treatment response in the respiratory system, it is very important to identify (i) whether the drug is delivered to the inflamed region and (ii) how much of the amount applied actually reaches the inflammation. Both are difficult to predict, especially when aerosols are applied. In a proof-of-principle experiment, the capability of dynamically capturing liquid instillation into the lung of a mouse was investigated and successfully demonstrated (Gradl *et al.*, 2019*a* ▸; Yang *et al.*, 2019 ▸). To this end, the benefit of PBI for generation of soft tissue contrast was combined with contrast-enhanced imaging in which the visibility of the tiny amount of liquid was increased by adding iodine-based contrast agent. However, sometimes projection imaging is not sufficient in order to determine material location, *e.g.* particle distributions in lungs or airways. In such a case, CT becomes necessary. Due to rapid degradation of biological substances like tissue, which would lead to motion artefacts in the reconstruction, these measurements have to be fast. As an example, a chest tomography of a recently deceased non-fixated mouse is shown in Fig. 8 ▸. 1500 projections were captured over 360° within 5 min, with 0.2 s exposure per projection and a continuous rotation. The air–tissue contrast was enhanced by 50 cm of free-space propagation and the corresponding edge-enhanced reconstruction is depicted in Fig. 8 ▸(*a*) and free of motion artefacts. Clear identification of bones and air-filled lung structures is possible. Applying the single-distance phase-retrieval algorithm (Paganin *et al.*, 2002 ▸) to the edge-enhanced data results in an improved differentiation between lung tissue and air, which is shown in Fig. 8 ▸(*b*). However, the homogeneity assumption (*i.e.* a constant ratio of δ and β for the whole specimen) within this algorithm introduces a slight blurring of the bony structures, since the ratio of δ and β was optimized for lung soft tissue (δ/β = 1891 at 25 keV). This ratio deviates from that of bones (δ/β = 156 at 25 keV). The ratios are calculated from the compositions of lung tissue and bone in Mohammadi *et al.* (2014 ▸). Fig. 8 ▸(*c*) is a volume rendering combined from both data sets. The lung rendering was performed using the phase-retrieved data set, while rib cage and beads (98 µm diameter) were rendered using the edge-enhanced one. Fig. 8 ▸(*d*) is the upper part of the left lung rendered from the phase-retrieved data set demonstrating the high resolution achievable for small airways in the fast CT scans. Additional motion renders acquisition of the same high-resolution tomography data *in vivo* more challenging. Often they suffer from poor resolution and/or long scan times [for example at the Skyscan small-animal scanner (Velroyen *et al.*, 2015 ▸)]. In particular, the respiratory system is challenging because the lung is a continuously moving organ, close to the beating heart. Here, we present such an *in vivo* CT of a mouse which was acquired within 15 min, *cf*. Fig. 9 ▸. The scan took longer in this case compared with the 5 min *ex vivo* one because image acquisition had to be synchronized to a short breath hold to minimize motion blur, which in turn resulted in a reduction of the frame rate from 5 frames s^−1^ to 1.1 frames s^−1^ due to the duration of the respiration cycle (including the breath hold) of 0.9 s. 1000 projections were taken over 360°. As the heart of the mouse is beating freely in this type of experiment, some motion blur is introduced. Moreover, fine airways and alveoli may still be displaced slightly in each image, even if a gating technique is used. Consequently, the spatial resolution is reduced in the *in vivo* case, Fig. 9 ▸, compared with the *ex vivo* case, Fig. 8 ▸.

The total time required for an *in vivo* propagation-based phase-contrast CT depends on the number of projections and thus the resolution, the ventilation frequency and the acquisition time for a single frame. These parameters can vary tremendously depending on the employed X-ray source and intention of the experiment. At synchrotrons and liquid-metal jet sources, single-frame acquisition times around 20 ms have been demonstrated for mechanically ventilated small-animal models (Stahr *et al.*, 2016 ▸; Murrie *et al.*, 2020 ▸). In these two set-ups, respiratory cycles lasted 0.45 s and 0.5 s during the acquisition of the CT. Consequently, data were taken at multiple positions in the respiratory cycle within the duration of a single CT scan, while only one frame per respiratory cycle was recorded in the proof-of-principle experiment at the MuCLS. For a sharp CT result, all projection images of a single CT have to be recorded at the same position in the respiratory cycle. Considering this boundary condition, a full tomography can be acquired about a factor of two faster with the reported parameters (Stahr *et al.*, 2016 ▸; Murrie *et al.*, 2020 ▸) compared with the MuCLS. However, pushing the total acquisition time to the smallest possible value was not the objective of the proof-of-principle experiment presented above. With the flux currently available at the MuCLS, acquisition times down to 30 ms offer sufficient image quality. This enables dynamic imaging of the respiratory cycle at the MuCLS similar to liquid-metal jet sources or synchrotrons. The final choice of the X-ray source for such measurements might be influenced by the attainable soft-tissue contrast and source availability. Synchrotron sources outperform both laboratory sources in the former, yet ICSs provide better soft-tissue contrast than liquid-metal jet sources (Töpperwien *et al.*, 2018 ▸). However, the acquisition time for a single frame might be slightly higher at the MuCLS.

### 5.4. Grating-based phase-contrast imaging   

**Figure 10:**
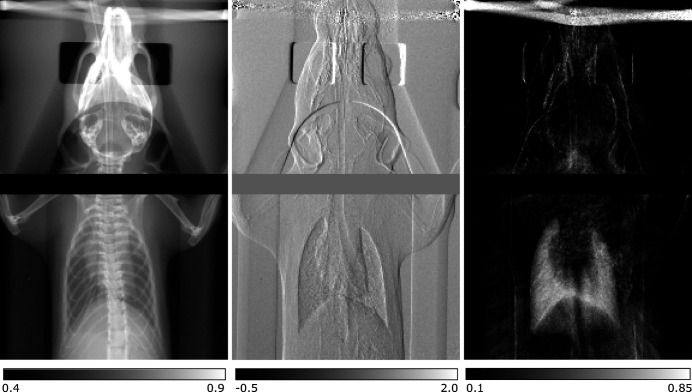
Projection images of an *in vivo* mouse obtained with grating-based imaging. On the left the absorption image is depicted, in the centre the differential phase-contrast one and on the right the dark-field image. Six images with individual exposure times of 140 ms were averaged improving the signal-to-noise ratio. The complementary information obtained by this technique can be seen very well, as different structures generate the most prominent contrast for the three different properties: bones in absorption, soft tissue in the differential phase contrast and dense scattering structures (*e.g.* lungs) in the dark field.

One drawback of PBI compared with grating-based imaging (GBI) with a quasi-monochromatic beam is that the former is not quantitative because the phase is typically reconstructed using the single material approximation. However, the most common implementation of GBI requires several images to be taken in order to extract the desired information and consequently is not suited for dynamics that require single-shot imaging, but may be applied for time-resolved imaging of a repeatable motion. A Talbot interferometer is employed in GBI which translates the object’s (differential) phase shift and the so-called dark-field signal, which can be related mainly to small-angle scattering (Bech *et al.*, 2012 ▸), into measurable intensity signals (Momose *et al.*, 2003 ▸; Weitkamp *et al.*, 2005 ▸; Pfeiffer *et al.*, 2008 ▸). GBI with an ICS has been demonstrated first by Bech *et al.* in 2009 (Bech *et al.*, 2009 ▸). Dynamic GBI with an ICS has been demonstrated very recently by resolving the respiration cycle of mice *in vivo* (Gradl *et al.*, 2019*b* ▸). This could enable identification of the best point in time for single-shot diagnostic lung imaging thereby reducing the dose significantly. Additionally, the dark-field signal ratio between exhalation and inhalation could be a non-invasive biomarker for local lung function allowing for better differentiation and detection of lung diseases, especially where alveoli are affected. The dynamic GBI capability is demonstrated in Fig. 10 ▸ for one point of the breathing cycle of an *in vivo* mouse. Image acquisition followed the procedure described by Gradl *et al.* (2019*b* ▸) with individual exposure times of 140 ms and averaging over six exposures. The complementary information of the three image modalities obtained by GBI, namely the absorption image on the left, differential phase-contrast image in the centre and dark-field image on the right, is very well visible. While bones show up prominently in absorption, this is the case for soft tissue, *e.g.* airways, in the differential phase-contrast image and structures with a high density of scattering structures, *e.g.* the lung, in the dark-field image. Furthermore, at the prototype of the Lyncean Compact Light Source, the dark-field signal has been demonstrated to be a good parameter for the diagnosis of emphysema in a pre-clinical study with *ex vivo* mice (Schleede *et al.*, 2012*b* ▸). A detailed overview over the different methods for dynamic respiratory X-ray imaging with living animals has been given by Morgan *et al.* (2020 ▸).

As mentioned earlier, (differential) phase-contrast imaging provides improved soft-tissue contrast. Therefore, phase contrast is expected to improve the diagnostic value of the images, *e.g.* in mammography. A first patient study was carried out at the Elettra synchrotron in Trieste, Italy, which demonstrated increased diagnostic value, but at the same time emphasized that the main limitation for a wide-spread use would be the availability of synchrotron radiation at clinics (Castelli *et al.*, 2011 ▸). Consequently, this is an interesting application for compact synchrotron sources, like the MuCLS. First, a phantom study was performed using GBI at a dose compatible with clinical mammography which yielded improved contrast over classical attenuation images (Schleede *et al.*, 2012*a* ▸). A follow-up study explored tomosynthesis at ICS (Eggl *et al.*, 2016*b* ▸). Very recently, GBI mammography at the MuCLS of mastectomy specimens has been shown to improve the delineation of tumorous lesions at an X-ray dose compatible with clinical mammography (Eggl *et al.*, 2018 ▸).

Combined with quasi-monochromatic X-rays, GBI allows for quantitative determination of the absorption coefficient as well as the phase shift. The complex refractive index can be reconstructed from this information, which enables quantitative material discrimination (Eggl *et al.*, 2015 ▸; Braig *et al.*, 2018 ▸). Therefore, this technique can be used for similar purposes as dual-energy imaging, while at the same time providing a reliable differentiation for both low-absorbing material, like soft tissue, as well as strongly absorbing materials, like bone or contrast agents. While the former vary mainly in electron density and consequently the real part of the refractive index, the latter exhibit considerable differences in their effective atomic number.

### 5.5. X-ray vector radiography and X-ray tensor tomography   

In the preceding discussion, the fact was omitted that gratings employed in GBI typically are line gratings. Consequently, wave modulation occurs in one direction only which generates a preferential sensitivity direction perpendicular to the grating lines for X-ray scattering as well as differential phase shifts. In order to employ this directional sensitivity for determination of structure orientations within the specimen, coined directional dark-field imaging, either the sample or the interferometer has to be rotated around the systems optical axis and images have to be acquired at each rotation position (Jensen *et al.*, 2010*a* ▸,*b* ▸). By these means, it is possible to determine the orientation of scattering structures with dimensions smaller than the detector resolution, *e.g.* fibrils in bones. We demonstrated at the MuCLS that micro-fractures, occult in classic radiography, can be detected by applying this technique which would be of diagnostic value in clinical practice (Jud *et al.*, 2017*a* ▸,*b* ▸). Nevertheless, this technique is not limited to (pre-)clinical diagnostics, but can also be applied in material science or quality assurance. Implementation of a three-circle Eulerian cradle expands this technique to three-dimensional reconstruction, so-called anisotropic X-ray dark-field tomography (AXDT) (Wieczorek *et al.*, 2016 ▸), the generalized form of X-ray tensor tomography (Malecki *et al.*, 2014 ▸). AXDT is currently not possible at the MuCLS, as the sample stage lacks one rotational degree of freedom, but is going to be available in the near future.

## 6. X-ray absorption spectroscopy   

X-ray absorption spectroscopy (XAS) is an element-selective probe of the surroundings of an atom of interest which provides information on its chemical state, geometric and electronic structures among others (Koningsberger & Prins, 1988 ▸). The main advantage of this technique compared with crystallography is that it can be applied to non-crystalline materials. At the MuCLS, an energy-dispersive set-up was implemented which exploits the provided X-ray bandwidth and divergence most efficiently. Due to the natural beam divergence of 4 mrad, the angle of incidence on a silicon wafer oriented in Bragg condition is spatially varying which translates into a spatially dependent energy of the diffracted beam. The sample is placed into the converging beam path and the X-rays are recorded by a 2D detector placed right after the sample, on which the X-ray energy is encoded in one spatial dimension. Since the X-rays penetrate the sample at different positions, a concentration correction is applied to the data. Details on the set-up, data acquisition and data processing have been given by Huang *et al.* (2020 ▸).

**Figure 11:**
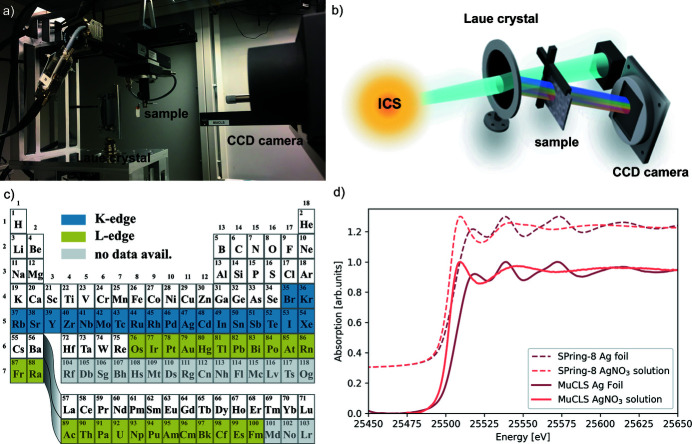
The XAS set-up is depicted in (*a*) with a schematic of the beam path shown in (*b*). The elements whose *K*- and *L*-edges can be covered by the energy range of the ICS of the MuCLS are highlighted in (*c*) in blue and green, respectively. Elements for which no data are available from the NIST database (NIST, 2005 ▸) are coloured in grey. As an example, a measurement of a silver foil and a silver nitrate solution is depicted in (*d*). For comparison, synchrotron measurements of the same substances are included and shifted upwards.

With this set-up located in the first experimental end-station and depicted in Figs. 11(*a*) and 11(*b*), X-ray absorption near-edge structure (XANES) and extended X-ray absorption fine structure (EXAFS) spectra have been collected at an energy resolution down to ∼4 eV at 25.5 keV. Examples of XANES spectra for a silver foil and a silver nitrate solution are depicted in Fig. 11 ▸(*d*). The total acquisition time for each of the spectra recorded at the MuCLS and depicted here was 10 min. Recently, data acquisition times as short as 1 min have been successfully demonstrated at a quality comparable with synchrotrons (Huang *et al.*, 2020 ▸). Other laboratory XAS set-ups, based on X-ray tubes (Seidler *et al.*, 2014 ▸; Németh *et al.*, 2016 ▸; Bès *et al.*, 2018 ▸; Błachucki *et al.*, 2019 ▸; Jahrman *et al.*, 2019 ▸), laser-plasma sources (Uhlig *et al.*, 2013 ▸), laser-betatron ones (Mahieu *et al.*, 2018 ▸) or high harmonic generation (Pertot *et al.*, 2017 ▸; Popmintchev *et al.*, 2018 ▸) typically operate at a lower X-ray energy below 10 keV and typically require exposure times of tens of minutes to hours. This elongated acquisition time originates from the lower brilliance of X-ray tubes compared with ICSs, like at the MuCLS. The difference in brilliance becomes more prominent at higher X-ray energies, which makes ICSs advantageous in this regime. In contrast to attosecond to few femtosecond pulses available from high harmonic generation or at laser-driven betatron sources, the pulse length at the MuCLS of ∼60 ps does not allow for ultrafast time-resolved spectroscopy. However, the X-ray energy range at the MuCLS allows to perform XAS measurements on a large range of the periodic table complementary to the typical sub-10 keV range of other laboratory XAS systems by probing either *K*-edges from bromine to xenon or *L*-edges from osmium at least to fermium, *cf*. Fig. 11 ▸(*c*). The absorption edge’s positions were retrieved from NIST (NIST, 2005 ▸). The energy range covered at once in the energy-dispersive XAS set-up is
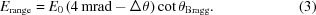

*E*_0_ denotes the central energy, θ_Bragg_ the corresponding Bragg angle and Δθ = *lR* ^−1^, where *l* is the diameter of the beam on the curved crystal and *R* is the crystal’s radius of curvature. In case the energy range is too small for the acquisition of a full EXAFS spectrum, it can be extended either by scanning the Bragg angle of the crystal in fine steps while recording images continuously or by stitching several exposures at more widely spaced Bragg angles. Switching to a different crystal plane which diffracts a larger energy range is another possibility. The energy resolution of the system can be approximated by
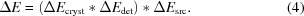
Δ*E*_cryst_ is the intrinsic energy resolution of the chosen crystal plane. Δ*E*_det_ is the contribution of the detector point-spread function which can be calculated from the energy resolution per pixel (determined by the energy bandwidth *E*_range_ and the number of illuminated pixels) and the detector point-spread function of the used camera, *cf*. Table 2 ▸. Δ*E*_src_ is the contribution of the X-ray source size which can be determined from the measured source size, typically ∼50 µm (σ-value), and the energy resolution per pixel. The * symbol denotes a convolution. Using these equations, the energy range and energy resolution can be optimized for the desired experiment.

## 7. Microbeam radiation therapy   

Finally, proof-of-principle studies on microbeam radiation therapy (MRT) have been performed at the MuCLS. Contrary to classical radiation therapy, where a spatially homogeneous dose is applied, the intensity is redistributed into microbeams with very high peak doses sparing tissue in between (Slatkin *et al.*, 1992 ▸). Brilliant X-rays are a requirement for this technique to work properly which means that MRT has been restricted to synchrotron facilities until now. Since ICSs provide a quasi-monochromatic, low-divergence beam and in principle are capable of producing very hard X-rays on a small footprint, they are well suited for this technique and currently seem to be the most promising option to transfer this technique into clinical practice in the long run. At the MuCLS, generation of microbeams with the required peak-to-valley dose ratio has been demonstrated at an X-ray energy of 25 keV (Burger *et al.*, 2017 ▸). This is much lower than typical energies of ∼100 keV used for studies at synchrotron facilities (Crosbie *et al.*, 2015 ▸). Treatment research at the MuCLS is therefore limited to superficial tumours, *e.g.* skin tumours. A dedicated set-up and small-animal model have been developed for this research. Details on the set-up are given by Burger *et al.* (2020 ▸) and about the small-animal model by Dombrowsky *et al.* (2020 ▸). At other ICSs like ThomX, which aims to generate higher X-ray energies, this limitation to superficial tumours does not apply.

## 8. Planned upgrades to the MuCLS beamline   

Although the MuCLS is extremely well suited for imaging applications, a few limitations have been identified which are currently addressed or are going to be addressed in the near future.

### 8.1. Grating-based imaging   

A stepper motor (LTA-HL; Newport Corp., Irvine, USA) in conjunction with a flexure-based nanoconverter developed at the Paul Scherrer Institute has been used for phase stepping in GBI (Henein *et al.*, 2007 ▸). Although this concept is very reliable, grating movement is inherently slow due to the employed stepper motor and because it is operated in uni-directional motion to achieve the desired position accuracy. As a consequence, dynamic imaging with the grating interferometer is limited by the motor movement overhead at the moment. Therefore, a new stepping mechanism was implemented recently by replacing the nanoconverter by a fast piezo-electric linear actuator. This speeds up motor movement for the distance of one step of the phase stepping by a factor of 18. Including typical acquisition times, communication overheads of the control system and detector readout, the speed-up with the piezo is still a factor of six to eight for typical phase-contrast tomography measurements compared with using the nanoconverter.

Thus, this new set-up is a significant improvement for future dynamic experiments, *e.g.* for *in vivo* respiratory dark-field imaging.

Furthermore, in Section 5.5 it was mentioned that AXDT is currently not available due to the missing third rotational degree of freedom. In order to overcome this limitation, a seven-axis robot arm (Panda; Franka Emika, Munich, Germany) was acquired and is currently being commissioned. The advantage of a robot arm over a conventional Eulerian cradle is that it can cover more angles without obstructing the X-ray beam itself. Moreover, it allows more flexible sample movement which might reduce data acquisition time for an AXDT measurement compared with one performed with an Eulerian cradle.

### 8.2. Replacement of the second end-station   

At the moment, the second end-station of the MuCLS offers only a very limited space for experiments. The main reason for this is that, at the time that this end-station was installed, another large laboratory X-ray set-up was located right next to it. Therefore, the X-ray hutch could neither be placed any closer to the source nor be wider as it would have blocked access to this other set-up. However, this other set-up was relocated to another laboratory recently in order to replace the existing end-station in 2021 with a new one which will be 6 m to 7 m long and 2.5 m to 3 m wide. This is going to enable access to a much larger range of beam diameters as well as much more flexible set-ups, thereby enhancing the MuCLS’s capabilities significantly.

## 9. Conclusion   

The Munich Compact Light Source, a laboratory-sized X-ray user facility based on an inverse Compton scattering X-ray source, has been presented. Inverse Compton scattering generates low-divergence partially coherent quasi-monochromatic, *i.e.* synchrotron-like, X-ray radiation on a laboratory scale. This enables the transfer of synchrotron techniques into university or industrial environments. The performance of the inverse Compton source installed at the MuCLS has been compared with state-of-the-art synchrotron sources as well as advanced X-ray tube sources. The versatile beamline at the MuCLS with two end-stations was described in detail including available detector options and design choices. The first end-station starts at a distance to the X-ray source of ∼3 m which makes it ideal for experiments requiring a smaller field of view but a high flux density. Among them are *in vivo* small-animal propagation-based phase-contrast imaging, micro-CT, X-ray absorption spectroscopy or microbeam radiation therapy research. The second end-station is downstream of the first one at a distance of ∼15 m to the X-ray source, where the beam’s coherence and field of view are larger. Consequently, large samples up to a diameter of ∼60 mm can be measured without stitching and for larger ones efficient stitching tools exist. A grating interferometer enables (quantitative) phase-contrast and dark-field imaging even of large specimens in this end-station, *e.g.* for mammography. Samples can be rotated around the X-ray beam to acquire information on the orientation of scattering structures via X-ray vector radiography. Another technique that can be used in both end-stations is *K*-edge subtraction imaging. All these techniques have already been successfully demonstrated at the MuCLS and are important tools in biomedical research and non-destructive testing. In combination with easier access compared with synchrotrons, especially for longitudinal studies, this opens up a wide field of applications for inverse Compton X-ray sources, like the MuCLS. 

## Figures and Tables

**Figure 12:**
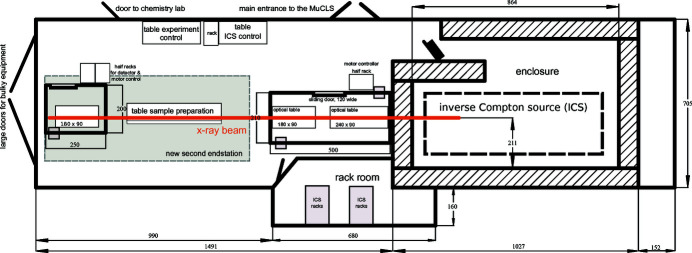
To-scale schematic of the MuCLS laboratory in the Munich School of BioEngineering of the Technical University of Munich.

**Table 1:** Summary of the current operating parameters of the X-ray source of the MuCLS

Repetition rate	64.91 MHz
Electron energy	29 MeV to 45 MeV
Storage ring current	16 mA
Electron pulse length	50 ps (Schleede, 2013 ▸)
Laser wavelength	1064 nm / 1.17 eV (Nd:YAG)
Laser power (inside the optical cavity)	∼ 350 kW
Laser pulse length	26 ps (FWHM)
X-ray energy	15 keV to 35 keV†
X-ray flux	Up to 1.5 × 10^10^ photons s^−1^ (15 keV)
	Up to 4.5 × 10^10^ photons s^−1^ (35 keV)
X-ray pulse length	60 ps (Feser *et al.*, 2018 ▸)
X-ray divergence	4 mrad
X-ray source size	∼ 50 µm (σ-value)
X-ray bandwidth	3% (15 keV) to 5% (35 keV) (FWHM)
X-ray brilliance	1.2 × 10^10^ photons s^−1^ (0.1% bandwidth)^−1^ mm^−2^ mrad^−2^

† The energy range in which Lyncean Technologie Inc. guarantees certain X-ray parameters.

**Table 2:** Overview of the camera systems available at end-station 1 The abbreviation PSF denotes point-spread-function, and FOV denotes field of view.

Model	Hamamatsu sCMOS-C12849-101U	Andor Zyla sCMOS 5.5	Ximea X-ray CCD – xiRay	Photonic Science 4.2MP sCMOS	Andor Neo sCMOS 5.5 (with 4×/10× optics)
Scintillator	Gd_2_O_2_S:Tb, 10 µm	Gd_2_O_2_S:Tb, 20 µm	Gd_2_O_2_S:Tb, 22 µm	Gd_2_O_2_S:Tb, 15 µm	LSO, 10 µm
Pixel size	6.5 µm	6.5 µm [13 µm]†	9 µm	11 µm	1.63 µm / 0.65 µm
PSF (σ)	8 µm	11 µm [60 µm]†	12 µm	11 µm	1.5–2 µm (10× optic)
FOV (pixel)	2048 × 2048	2560 × 2160	4008 × 2672	2048 × 2048	2560 × 2160
FOV (mm)	13.3 × 13.3	16.6 × 14.0	37.3 × 25.7	22.5 × 22.5	4.17 × 3.52 /
		[33.3 × 28.0]†			1.66 × 1.40
Maximum frames s^−1^	30 frames s^−1^	100 frames s^−1^	2.1 frames s^−1^	18 frames s^−1^	100 frames s^−1^
Dynamic range	18000:1, 15 bit	12500:1, 14 bit	6000:1, 13 bit	38800:1, 16 bit	13000:1, 14 bit
Digitization	16 bit	16 bit	14 bit	16 bit	16 bit

† Values for the optional 2:1 fibre-optic taper available for the Andor Zyla sCMOS 5.5.

**Table 3:** Overview of the camera systems available at end-station 2 The abbreviation PSF denotes point-spread function and FOV denotes field of view.

Model	Perkin Elmer Dexela 1512	Dectris Santis (evaluation prototype)	Direct Conversion XC-Thor	Varian PaxScan 2520D CsI	Dectris Pilatus 200 K
Scintillator / sensor	Gd_2_O_2_S:Tb, 150 µm	GaAs, 500 µm	CdTe, 750 µm	CsI, 600 µm	Si, 1000 µm
Pixel size	74.8 µm	75 µm	100 µm	127 µm	172 µm
PSF	71 µm (σ)	75 (box) µm	100 (box) µm	NA	172 (box) µm
FOV (pixels)	1536 × 1944	1030 × 1064	1024 × 512	1536 × 1920	487 × 407
FOV (mm)	114.9 × 145.4	77.25 × 79.80	102.4 × 51.2	195 × 243.8	83.7 × 70.0
Maximum frame rate	26 frames s^−1^	25 frames s^−1^	300 frames s^−1^	10 frames s^−1^	20 frames s^−1^
Dynamic range	2000:1, 12 bit	65536:1, 16 bit for each counter	4096:1, 12 bit	NA	1048573:1, 20 bit
Digitization	14 bit	32 bit	12 bit	16 bit	20 bit

**Table 4:** Parameters of the gratings installed in the interferometer in end-station 2

Grating	G1 (25 keV)	G1 (35 keV)	G2
Period	4.92 µm	4.92 µm	5 µm
		4.89 µm at 6.3°	
Height	4.39 µm	6.15 µm	>70 µm
Material	Ni	Ni	Au
Type	π/2 phase-shift	π/2 phase-shift	Absorption
Duty cycle	0.5	0.5	0.5
Geometry	70 mm diameter	70 mm diameter	70 mm diameter
Substrate	525 µm Si	525 µm Si	525 µm Si

## Appendix A Schematic of the MuCLS laboratory

A to-scale sketch of the MuCLS laboratory is depicted in Fig. 12 ▸. Only the experimental hall housing the MuCLS is depicted; other laboratories and offices are omitted. The size of the whole laboratory is 26.70 m × 7.05 m × 4.6 m, of which the enclosure of the ICS itself occupies a volume of 10.27 m × 7.05 m × 3.34 m. Its walls are ∼80 cm thick and realized as a sandwich structure of heavy concrete filled with electro-furnace slag. The door of the enclosure is 0.9 m wide, which is sufficient to move a modulator out of the cave, *e.g.* in order to replace its klystron. The small area to the right of the enclosure in Fig. 12 ▸ houses the three chillers of the ICS, while most of the machine instrumentation, especially magnet power supplies and RF control systems, are located in a separate rack room which contains an additional forced-air cooling unit removing the heat dissipated mainly by the magnet power supplies. The ICS control as well as beamline control are located next to each other in the laboratory. Both end-stations can be controlled independently from each other enabling preparation of experiments in the second end-station while measurements are running in the first one. Moreover, workbenches for sample preparation are located between both end-stations with a chemical laboratory next door for advanced preparation protocols. The area that is coloured in ivory indicates the approximate extend of the new second end-station which is scheduled to replace the small one in 2020.
